# A Novel Conical Spiral Transmission Line Sensor-Array Water Holdup Detection Tool Achieving Full Scale and Low Error Measurement

**DOI:** 10.3390/s19194140

**Published:** 2019-09-24

**Authors:** Yong Wei, Houquan Yu, Qiang Chen, Guoquan Liu, Chaoxian Qi, Jiefu Chen

**Affiliations:** 1 School of Electronics and Information, Yangtze University, Jingzhou 434023, China; 2 Equipment and Sales Branch, China Petroleum Logging CO. LTD., Xi’an 710077, China; 3 Department of Electrical and Computer Engineering, University of Houston, Houston, TX 77044, USA

**Keywords:** oil–water two-phase flow, water holdup, conical spiral transmission line, sensor, electromagnetic wave, horizontal well

## Abstract

To dynamically monitor the horizontal well, we studied the oil–water two-phase water holdup detection method based on transmission lines, and designed a micro-sensor and a sensor-array water holdup detection tool. We modeled the relationship of the dielectric constant of the transmission line filling medium and the amplitude and phase shift of the electromagnetic wave signal on the transmission line by using the time-domain analysis. We proposed a novel method to measure the water holdup of oil–water mixtures based on the phase shift of signals on the conical spiral transmission line. Furthermore, we simulated and optimized the structural parameters by software simulation, and developed a small conical spiral water holdup sensor suitable for arraying. The single sensor with detection circuits can achieve the full scale (water holdup from 0% to 100%) measurement with resolution better than 3%. On this basis, 12 sensors are used to develop a clock-like sensor-array water holdup detection tool, realizing the array detection of the distribution of the cross-section medium in horizontal wells.

## 1. Introduction

The rapid expansion of the horizontal well application and the waterflooding technology has lead to more serious reservoir water problems. The most effective method to measure the distribution and location of water production is to detect water holdup, which can directly affect the production efficiency of horizontal wells. Although there are many methods for water holdup detection at present, only a few tools can really be used to detect water holdup in horizontal wells due to the limitation of high temperature, high pressure and narrow space. For example, the Flow Scanner [1] produced by Schlumberger and Capacitance Array Tool [2] and the Resistance Array Tool [3,4] produced by Sondex. Their detection methods can be basically divided into the conductance method and the capacitance method.

The conductance method is based on the huge differences in oil and water conductance. Generally, the water holdup is estimated by the measured conductance of the fluid. In the past 10 years, Jin’s research team designed conductance sensors with various structures and carried out according experimental studies [5,6,7,8]. However, the conductance method is affected by fluid salinity, and in particular, it will fail if the water holdup is less than 20% because there is no continuous water phase between measuring electrodes. The capacitance method [9,10,11,12] is based on the monotonically increasing relationship between water holdup and dielectric constant of oil–water two-phase fluid. The fluid flows between the two poles of the capacitor and plays as a filling medium, thus the water holdup measurement is transformed as the capacitance measurement. The main reasons why the capacitance method loses the resolution is the influence of groundwater salinity. In fact, the change of resistance caused by groundwater salinity can be regarded as a resistor parallel to the capacitor sensor. The increase of salinity will increase the proportion of the conduction current on the resistor, while the proportion of displacement current on the capacitor caused by oil–water dielectric constant will decrease. When salinity reaches a certain level, the measurement error of the capacitance method will increase until the method fails.

According to the theory of electromagnetic wave propagation [13], the propagation characteristics of a transmission line are related to the dielectric constant and conductivity of the medium between its conductors. When the dielectric constant or conductivity of the medium changes, the amplitude and phase of the signal on the transmission line will change accordingly. It can be inferred that if the downhole fluid is used as a filling medium between the transmission lines, the characteristics of the signals on transmission lines will change when the water holdup of the fluid changes.

In 1993, Guo et al. [14] measured the water holdup by using the coaxial transmission line (CTL) with an open terminal. The idea was that the CTL filled with oil and water was regarded as a coaxial capacitor, and the resonant frequency of the circuit would change with the change of water holdup. The water holdup could be calculated through the resonant frequency. In 2002, Wang et al. [15] designed a CTL sensor, the frequency of the electromagnetic wave is 75 MHz, but the influence of salinity still exists. Meng et al. [16,17,18] optimized the CTL sensor and pointed out that the attenuation of the electromagnetic wave signal caused by salinity can be reduced by wrapping an insulating medium on the surface of the inner conductor of the sensor. On this basis, Yu et al. [19,20] increased the frequency of the signal appropriately and designed a water holdup detection tool based on CTL. Since the fluid flows between the inner and outer conductors of the CTL, the size of the sensor cannot be too small to avoid blockage. Because water holdup at the same cross section in a vertical well is basically the same, it is feasible to use a single point measurement method with a sensor. Therefore, the CTL sensor is suitable for water holdup measurement in vertical wells.

Due to gravity, oil and water in horizontal wells form a stratified flow, and thus a single sensor with a CTL structure can only measure the local area, and cannot obtain global information. Therefore, we need to use a multiple sensors array to detect the distribution of oil and water, so as to calculate water holdup. The key to this is the miniaturization of the sensor. Huo et al. [21,22] adopted a double CTL to measure the water holdup, which reduced the size of the sensor. Mohamed et al. [23] proposed a coplanar microstrip transmission line sensor with an “n” structure. On this basis, Wei et al. [24,25,26] designed a coplanar waveguide (CPW) sensor, using “s” type double-sided wiring to increase the length of the transmission line in limited space. Although the CPW sensor has good sensitivity, it easily adheres to liquid on the surface due to the flat structure and the influence of surface tension, and especially when the plane of the sensor is parallel to the horizontal plane, a measurement error may be caused.

Based on the above research, it is necessary to further study the propagation characteristics of signals on transmission lines and optimize the structure of the sensor. In this study, first, a water holdup detection model based on a general transmission line is established. Then, a novel conical spiral transmission line sensor (CSTLS) is proposed based on the requirements of the water holdup array detection in the horizontal well. The CSTLS parameters are optimized by studying the influence of the structure on the phase shift output. Experimental results show that the sensor can achieve the full-scale measurement of well water holdup from 0% to 100% with a resolution that is better than 3%. Finally, a sensor-array water holdup detection tool based on multi-CSTLS is developed, which realizes the array detection of the fluid distribution of the cross-section in horizontal wells.

## 2. Method

### 2.1. Propagation Characteristics of Signals on the Transmission Line

**Figure 1:**
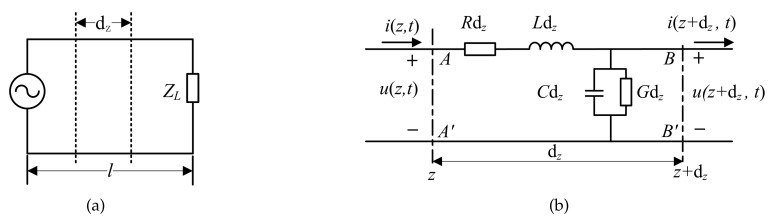
Equivalent circuit model of the transmission line: (**a**) Basic circuit model; (**b**) Lumped parameter circuit model.

According to electromagnetic wave propagation theory [27,28,29,30], the propagation characteristics of the signal on the transmission line were determined by the unit resistance and inductance of transmission line conductors, as well as the unit capacitance and conductance of the medium between transmission line conductors. If the medium is changed, its dielectric constant and conductivity will change, causing the change of the distribution parameters of transmission lines, as well as the change of their transmission signal characteristics. Therefore, when an oil–water mixture is used as the filling medium between transmission line conductors, the dielectric constant and conductivity information of the medium can be obtained by measuring the propagation characteristics of the signal, and then the water holdup of the oil–water mixture can be estimated accordingly. Thus, we studied the propagation characteristics of signals in transmission lines. The equivalent circuit of the transmission line is shown as Figure 1 under the transmitting TEM wave.

Figure 1a is the basic circuit model of the transmission line, in which a conductor of infinitesimal length dz can be simulated by the lumped parameter circuit model in Figure 1b. *R* is the resistance per unit length (Ω/m), *G* is the conductance per unit length (S/m), *L* is the inductance per unit length (H/m), and *C* is the capacitance per unit length (F/m). If the voltage and current at the beginning of d${}_{z}$ (position $AA^{\prime }$) are *u*(*z*, *t*) and *i*(*z*, *t*), respectively, and the voltage and current at the end of d${}_{z}$ (position $BB^{\prime }$) are *u*(*z* + d${}_{z}$, *t*) and *i*(*z* + d${}_{z}$, *t*), respectively. According to Kirchhoff’s law, there is

(1) $$\left\{\begin{array}{c} \frac{d^{2}u\left(z\right)}{dz^{2}}=ZYu\left(z\right)=\gamma^{2}u\left(z\right)\\ \frac{d^{2}i\left(z\right)}{dz^{2}}=YZi\left(z\right)=\gamma^{2}i\left(z\right) \end{array}\right.$$

where, Z=R+jωL, Y=G+jωC, γ is the propagation constant, $\gamma =\sqrt{\left(R+j\omega L)(G+j\omega C\right)}=\alpha +j\beta$, α and β are the amplitude attenuation factor and phase shift factor per unit length of the transmission line, respectively.

The general solution of the differential Equation (1) is:

(2) $$U\left(z\right)=Ae^{\gamma z}+Be^{-\gamma z}$$

(3) $$I\left(z\right)=\frac{1}{Z}\frac{dU(z)}{dz}=\frac{1}{Z_{0}}\left(Ae^{-\gamma z}-Be^{\gamma z}\right)$$

where, $Z_{0}=\sqrt{\left(Z/Y\right)}=\sqrt{\left(R+j\omega L)/(G+j\omega C)\right)}$, which is the characteristic impedance of the transmission line. The undetermined constants *A* and *B* need to be determined according to the port conditions.

If the voltage $U\left(0\right)=U_{2}$ and current $I\left(0\right)=I_{2}$ are known in the terminal (z=0), then there are $A=(U_{2}+Z_{0}I_{2})/2$, $B=(U_{2}-Z_{0}I_{2})/2$. Substitute them into Equations (2) and (3), as follows:

(4) $$U\left(z\right)=U_{2}^{+}e^{\gamma z}+U_{2}^{-}e^{-\gamma z}$$

(5) $$I\left(z\right)=\frac{1}{Z_{0}}\left(U_{2}^{+}e^{\gamma z}+U_{2}^{-}e^{-\gamma z}\right)$$

where, $U_{2}^{+}=\left(U_{2}+I_{2}Z_{0}\right)/2$, $U_{2}^{-}=\left(U_{2}-I_{2}Z_{0}\right)/2$.

Equations (4) and (5) are the expressions of voltage and current at any point on the transmission line when the terminal voltage and current of the transmission line are known. The two equations show that the voltage and current on the transmission line exist in the form of waves and are composed of two parts: the transmitting signal and the reflecting signal. If the ratio of the reflected signal voltage to the transmitted signal voltage at a point *z* on the transmission line is defined as the reflection coefficient Γ(z) at that point, then

(6) $$\Gamma \left(z\right)=\frac{U^{-}}{U^{+}}=\frac{\left(U_{2}-Z_{0}I_{2}\right)e^{-\gamma z}}{\left(U_{2}+Z_{0}I_{2}\right)e^{\gamma z}}=\Gamma_{2}e^{-2\gamma z}$$

where, $\Gamma_{2}$ is the terminal reflection coefficient of the transmission line, and

(7) $$\Gamma_{2}=\frac{U_{2}-Z_{0}I_{2}}{U_{2}+Z_{0}I_{2}}=\frac{Z_{L}-Z_{0}}{Z_{L}+Z_{0}}$$

Therefore, according to the difference of terminal reflection coefficient, there are three different transmission modes of signals on transmission lines, namely, matching state, standing wave state and mixed wave state [13].

### 2.2. General Expressions of the Amplitude and Phase Shift of Signals on Transmission Line in Mixed Wave Mode

As the dielectric constant of the oil–water mixture fluid flowing between the conductors of the transmission line varies randomly, the characteristic impedance of the transmission line is a function of the dielectric constant of the mixture fluid. Therefore, when the terminal load of the transmission line is constant, the transmission line generally works in the mixed wave state rather than the matching state, unless the load adaptively matches the characteristic impedance. Therefore, in order to use the electromagnetic wave characteristics on the transmission line to detect the dielectric constant of oil–water fluid, it is necessary to study the general expressions of electromagnetic wave amplitude and phase shift at the end of the transmission line in the mixed wave state.

In order to study the signal characteristics of the transmission line terminals, assuming that the voltage $U\left(l\right)=U_{1}$ and the current $I\left(l\right)=I_{1}$ at the transmitter are known, the constants *A* and *B* can be determined by Equations (2) and (3), respectively.

(8) $$A=\frac{U_{1}+I_{1}Z_{0}}{2}e^{-\gamma l},B=\frac{U_{1}-I_{1}Z_{0}}{2}e^{\gamma l}$$

Substitute it into Equation (2), the voltage at any point *z* on the transmission line is

(9) $$U\left(z\right)=\frac{U_{1}+I_{1}Z_{0}}{2}e^{-\gamma \left(l-z\right)}+\frac{U_{1}-I_{1}Z_{0}}{2}e^{\gamma \left(l-z\right)}$$

Let $U_{1}^{+}=\left(U_{1}+I_{1}Z_{0}\right)/2$, $U_{1}^{-}=\left(U_{1}-I_{1}Z_{0}\right)/2$, the terminal voltage (z=0) can be expressed as

(10) $$U\left(0\right)=U_{1}^{+}e^{-\gamma l}+U_{1}^{-}e^{\gamma l}$$

If the reflection coefficient $\Gamma_{2}$ is known, the above formula can be expressed as

(11) $$U\left(0\right)=U_{1}^{+}e^{-\gamma l}+\Gamma_{2}U_{1}^{+}e^{\gamma l}=U_{1}^{+}\left(e^{-\gamma l}+\Gamma_{2}e^{\gamma l}\right).$$

Equation (11) is the expression of the relationship between the transmitting end voltage and the receiving end voltage. Therefore, the ratio of the receiving end voltage to the transmitting end voltage is the transfer function H(jω) of the transmission line, i.e.

(12) $$H\left(j\omega \right)=\frac{U(0)}{U_{1}^{+}}=e^{-\gamma l}+\Gamma_{2}e^{\gamma l}=e^{-(\alpha +j\beta )l}+\Gamma_{2}e^{(\alpha +j\beta )l}=cos\beta l\left(e^{-\alpha l}+\Gamma_{2}e^{\alpha l}\right)+jsin\beta l\left(\Gamma_{2}e^{\alpha l}-e^{-\alpha l}\right).$$

Equation (12) can be rewritten as H(jω)=Re(H(jω))+jIm(H(jω)), where Re(H(jω)) and Im(H(jω)) are the real and imaginary parts of the transfer function respectively, and

(13) $$Re\left(H\left(j\omega \right)\right)=cos\beta l\left(\Gamma_{2}e^{\alpha l}+e^{-\alpha l}\right),Im\left(H\left(j\omega \right)\right)=sin\beta l\left(\Gamma_{2}e^{\alpha l}-e^{-\alpha l}\right).$$

Assuming that the voltage at the transmitting end of the transmission line is $A_{0}sin\left(\omega t\right)$, the voltage at the receiving end is $U\left(0\right)=A_{am}sin\left(\omega t-\phi \right)$, then

(14) $$A_{am}=\left|H(j\omega )\right|A_{0}=A_{0}\sqrt{Re^{2}\left(H\left(j\omega \right)\right)+Im^{2}\left(H\left(j\omega \right)\right)}=A_{0}\sqrt{{\left(\Gamma_{2}e^{\alpha l}+e^{-\alpha l}\right)}^{2}-4\Gamma_{2}sin^{2}\beta l}.$$

Compared with the transmitting end, the phase shift of the signal at the receiving end of the transmission line is Φ, and

(15) $$\Phi =arctan\frac{Im(H(j\omega ))}{Re(H(j\omega ))}=arctan\frac{sin\beta l(e^{-\alpha l}-\Gamma_{2}e^{\alpha l})}{cos\beta l(e^{-\alpha l}+\Gamma_{2}e^{\alpha l})}=arctan\left(tan\left(\beta l\right)\frac{e^{-\alpha l}-\Gamma_{2}e^{\alpha l}}{e^{-\alpha l}+\Gamma_{2}e^{\alpha l}}\right).$$

According to the definition of reflection coefficient, Equations (14) and (15) can be rewritten as

(16) $$A_{am}=A_{0}\sqrt{\frac{{\left(Z_{L}-Z_{0}\right)}^{2}e^{2\alpha l}+{\left(Z_{L}+Z_{0}\right)}^{2}e^{-2\alpha l}+2\left(Z_{L}^{2}-Z_{0}^{2}\right)\left(cos^{2}\beta l-sin^{2}\beta l\right)}{{\left(Z_{L}+Z_{0}\right)}^{2}}}$$

(17) $$\Phi =arctan\left(\frac{\left(Z_{L}-Z_{0}\right)e^{\alpha l}-\left(Z_{L}+Z_{0}\right)e^{-\alpha l}}{\left(Z_{L}-Z_{0}\right)e^{\alpha l}+\left(Z_{L}+Z_{0}\right)e^{-\alpha l}}tan\left(\beta l\right)\right).$$

Equations (16) and (17) are general expressions of the amplitude and phase shift of the terminal signal when the transmission line is in a lossy mode. When the transmission line is in a lossless mode, that is, the unit resistance R=0, the unit conductance G=0, and the attenuation factor α=0, the above equations can be simplified as

(18) $$A_{am}=\frac{2A_{0}\sqrt{Z_{L}^{2}cos^{2}\beta l+Z_{0}^{2}sin^{2}\beta l}}{\left(Z_{L}+Z_{0}\right)}$$

(19) $$tan\Phi =\frac{Z_{0}}{Z_{L}}tan\left(\beta l\right)=\frac{Z_{0}}{Z_{L}}tan\left(l\omega \sqrt{LC}\right)=\frac{Z_{0}}{Z_{L}}tan\left(l\omega \sqrt{LK_{c}\varepsilon_{r}}\right)$$

where, *L* and *C* are the equivalent inductance and capacitance of the transmission line, respectively; $\varepsilon_{r}$ is the dielectric constant of the medium between the conductors of the transmission line and $K_{c}$ is the proportional coefficient between *C* and $\varepsilon_{r}$. In particular, when the load impedance is matching with characteristic impedance, $Z_{L}=Z_{0}$, then, $A_{am}=A_{0}$, $\Phi =\beta l=l\omega \sqrt{LK_{c}\varepsilon_{r}}$, and there is only incident wave and no reflected wave at the end of the transmission line.

In summary, (1). In the matching state, the phase shift of the signal is determined by the factor β. The larger the β value, the larger the phase shift of the signal on the transmission line. The β is related to the conductivity and dielectric constant of the medium; (2). In mixed wave state, the amplitude attenuation is determined by the factor α. The larger the α, the greater the attenuation of the signal on the transmission line. The α is related to the conductivity and dielectric constant of the medium; (3). In mixed wave state, if the terminal load and the length of the transmission line are constant, the amplitude and phase characteristic of the terminal signal are functions of the attenuation factor α, the phase shift factor β, and the characteristic impedance $Z_{0}$.

### 2.3. Principle of Detecting the Dielectric Constant of an Oil–Water Mixture Based on the Transmission Line

**Figure 2:**
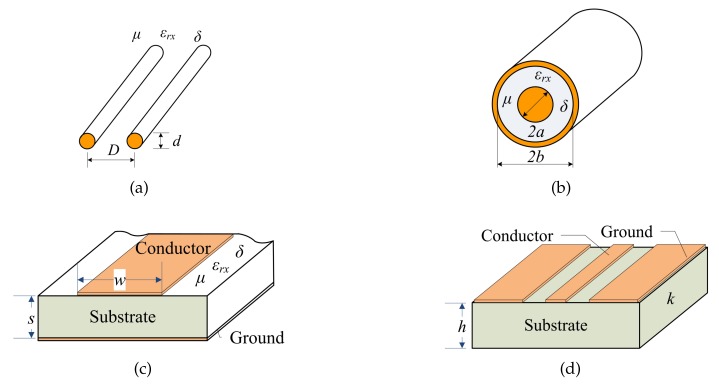
Four common transmission lines: (**a**) Parallel line; (**b**) Coaxial cable; (**c**) Microstrip; (**d**) Coplanar microstrip lines.

Based on the above discussion, if we can prove that the amplitude or phase shift of the signal on the transmission line in the mixed wave mode has a monotonic relationship with the dielectric constant of the medium between transmission lines, we can provide a feasible method to detect water holdup in oil–water mixtures. Figure 2 is the structure diagram of four commonly used transmission lines. We assume that the conductivity of oil, water and their mixture is zero under ideal conditions. Table 1 shows the relationship between the characteristic impedance $Z_{0}$ and the phase shift factor β of four transmission lines and the dielectric constant when the oil–water mixture is used as the filling medium under ideal conditions.

Table 1 shows that $Z_{0}$ is inversely proportional to $\sqrt{\varepsilon_{0}\varepsilon_{r}}$, and β is proportional to $\sqrt{\varepsilon_{0}\varepsilon_{r}}$. Therefore, $Z_{0}$ and β can be rewritten as the following general expressions, respectively:

(20) $$Z_{0c}=\frac{C_{z}}{\sqrt{\varepsilon_{eff}}},\beta_{c}=\omega C_{\beta }\sqrt{\varepsilon_{eff}}$$

where, $C_{z}$ and $C_{\beta }$ are constants, depending on the structure of the transmission line. Substitute (20) into (18) and (19), there are

(21) $$A_{am}=\frac{2A}{C_{z}/\sqrt{(\varepsilon_{eff}})+Z_{L}}\sqrt{C_{z}^{2}sin^{2}\left(C_{\beta }\omega l\sqrt{\varepsilon_{eff}}\right)/\varepsilon_{eff}+Z_{L}^{2}cos^{2}\left(C_{\beta }\omega l\sqrt{\varepsilon_{eff}}\right)}$$

(22) $$\Phi =sin^{-1}\left(\frac{C_{z}sin\left(C_{\beta }\omega l\sqrt{\varepsilon_{eff}}\right)}{\sqrt{C_{z}^{2}sin^{2}\left(C_{\beta }\omega l\sqrt{\varepsilon_{eff}}\right)+Z_{L}^{2}\varepsilon_{eff}cos^{2}\left(C_{\beta }\omega l\sqrt{\varepsilon_{eff}}\right)}}\right)$$

In order to further analyze the relationship between the amplitude and phase shift of the terminal signal on the transmission line and dielectric constant, we carry out digital simulation. When the load $Z_{L}$ is different, the relationship between amplitude and phase shift and dielectric constant is numerically simulated by (21) and (22).

**Figure 3:**
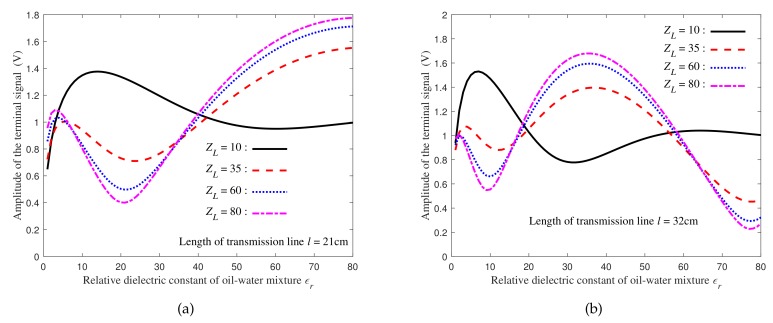
Relationships between the terminal voltage amplitude and the dielectric constant of two length transmission lines under different load conditions: (**a**) The length of the transmission line is 21 cm; (**b**) The length of the transmission line is 32 cm.

Figure 3 shows that the relationship between voltage amplitude and dielectric constant is not monotonic, regardless of the length of transmission line or the load of transmission line. This non-monotonicity leads to the uncertainty of water holdup inversion according to the measured signal voltage amplitude.

**Figure 4:**
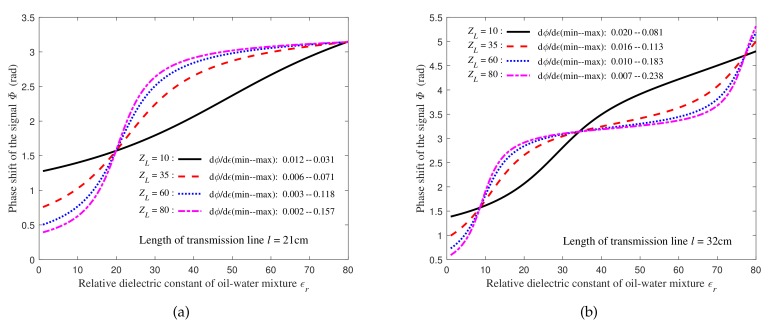
Relationships between the terminal signal phase shift and the dielectric constant of two length transmission lines under different load conditions: (**a**) The length of the transmission line is 21 cm; (**b**) The length of the transmission line is 32 cm.

Figure 4 shows that the phase shift of the signal is monotonically increasing with the dielectric constant of the medium. Therefore, it is theoretically feasible to estimate the dielectric constant of the medium around the transmission line by detecting the phase shift of the signal on the transmission line.

### 2.4. Sensor Requirements for Sensor-Array Water Holdup Detection Tool in Horizontal Wells

**Figure 5:**
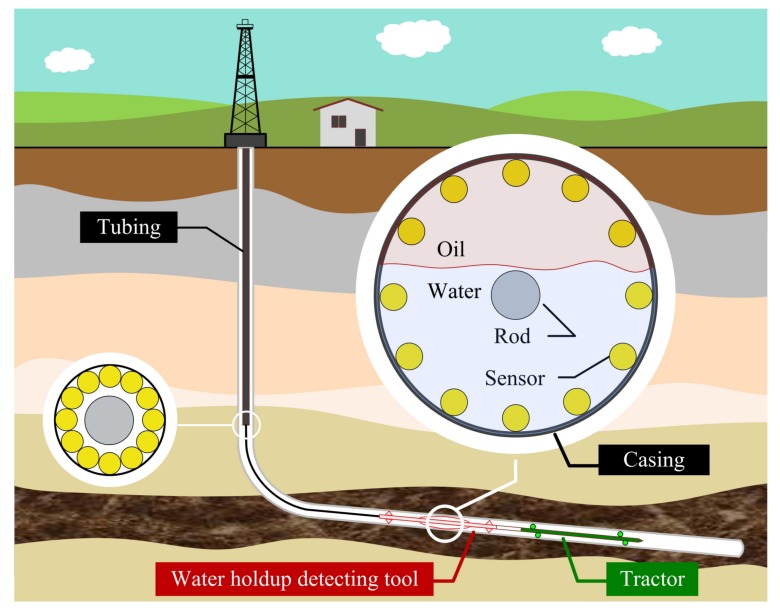
Deployment diagram of array water holdup sensors.

The sensor-array water holdup detection tool has two modes: moving and logging. The moving mode is mainly the process of lifting and lowering the tool in the casing. Due to different well conditions, it is sometimes necessary to pass the tool through a 2 and 3/8 inch diameter tubing. This requires that the maximum diameter of the tool should not be greater than 43 mm when all sensors are gathered, to ensure that the tool slides freely in the tubing, as shown on the left side of Figure 5. The logging mode is the measuring state when the tool reaches the target layer. At this point, the sensor is close to the casing under the action of spring tension, and the maximum diameter of the tool is slightly less than the inner diameter of the casing. If the outer diameter of the casing is 7 inches, the diameter of the spring after opening is about 160 mm, as shown on the right side of Figure 5.

According to the structure and stiffness requirement, the radius of the central support rod of the tool should be greater than 4 mm. If the thickness of the fixed bow spring is 1 mm, the space reserved for sensors is actually an annular space with an inner radius no less than 4 mm and an outer radius no more than 20.5 mm. If these sensors are distributed on the same cross-section of the tubing during the moving mode, they are gathered into a circumscribed circle. Therefore, the maximum outer diameter of each sensor should be less than 8 mm. This also minimizes the influence of the sensor on the flow pattern of the fluid. If the flow direction resolution of the tool is further considered, the effective length of the sensor should not exceed 80 mm. Therefore, the overall size of the sensor is limited to a cylindrical space with a diameter of 8 mm and a length of 80 mm.

In summary, the design requirements of the array water holdup sensor are as follows: (1) small section (diameter ⩽ 8 mm, which is convenient for arranging multiple sensors on the well section to form a detection array); (2) short length (length ⩽ 80 mm, which makes it easy to improve flow direction resolution); (3) high precision (resolution up to 3%, for maintaining high precision over the whole scale).

### 2.5. Structure and Numerical Simulation of Cstls

Firstly, we discuss whether a common CTL is suitable for sensor design. In order to ensure the sensor strength, the radius of the inner and outer conductors of the CTL cannot be less than 1 mm. If the diameter of the sensor is less than 8 mm, then the fluid annulus in the CTL is less than 2 mm. Such a narrow space can cause fluid flow obstruction. Therefore, the CTL cannot meet the space requirements, and we should look for other solutions.

CPW is very easy to miniaturize, and we have previously designed an S-type CPW sensor [24,25,26]. However, because of surface tension, the oil–water mixture easily adheres to sensor surfaces with a planar structure, thus affecting the sensitivity. In order to reduce liquid adsorption, we propose the CSTLS, which is changed from a planar structure to a conical spiral structure.

**Figure 6:**
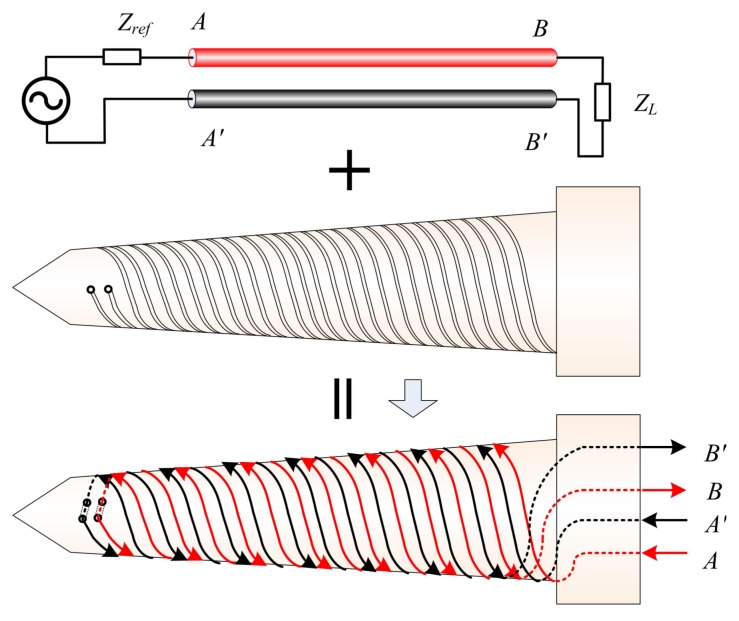
Schematic of a conical spiral transmission line sensor (CSTLS).

As shown in Figure 6, the basic principle of CSTLS is similar to the parallel line. The specific design procedure is as follows: First, a cone is machined with PEEK (polyetherether ketone) material, and four spirals are cut out on the cone. Second, two enameled wires are rotated from the bottom of the cone to the top of the cone, then passed through two holes on the top of the cone, and then circled back to the bottom of the cone, thus forming CSTLS.

**Figure 7:**
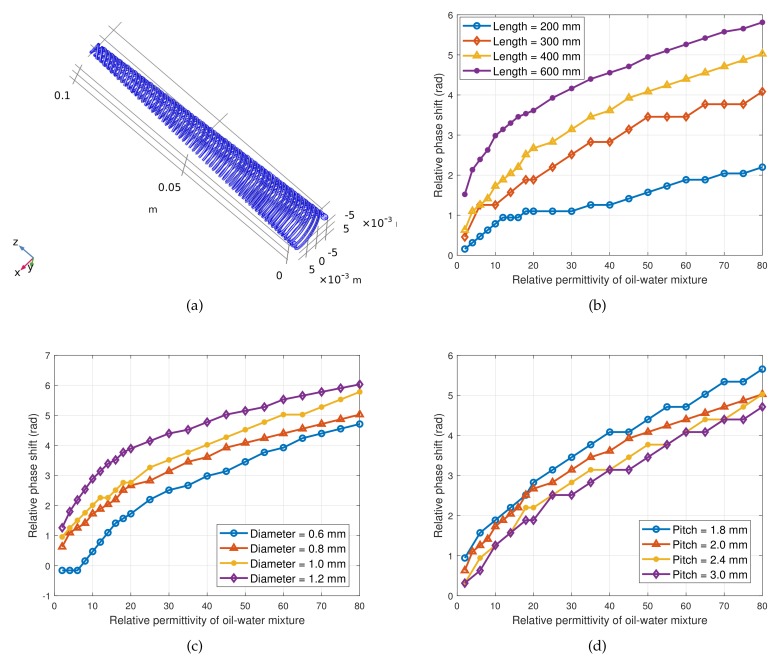
Relationship between structural parameters of CSTLS and Φ: (**a**) Model; (**b**) The effect of equivalent length of CSTLS on phase shift; (**c**) The effect of wire diameter of CSTLS on phase shift; (**d**) The effect of pitch of CSTLS on phase shift.

In order to design the sensor reasonably, we need to analyze the influence rule of the sensor structure on the phase shift to provide theoretical guidance for selecting appropriate structure parameters. From winding direction (A→B), we can find that the coil diameter first decreases continuously, and then increases continuously. This irregular structure makes it difficult to model the distribution parameters of the transmission line using traditional methods. Therefore, even if we have derived Equation (22), it is difficult to obtain the exact analytical formula for the relative dielectric constant $\varepsilon_{rx}$ of the oil–water mixture and the structure parameters with the signal phase shift Φ. However, the powerful simulation function of COMSOL(a multiphysics modeling software) can help us determine these irregular physical fields. We have known that the closer the linear relationship between Φ and $\varepsilon_{rx}$ is, the more consistent the sensitivity is; the greater the variation in Φ in the full scale (not exceeding 2π), the higher the resolution of the sensor. Based on this, we built a model and simulate it using COMSOL. When investigating the influence of a certain parameter on Φ, four different values were selected and the rest of the parameters were selected by default as shown in Table 2. The simulation results are shown in Figure 7.

Figure 7a is the simulation model; Figure 7b shows that the longer the transmission line is, the larger the dynamic range of Φ, but it should be limited within a certain range of the length to ensure that the maximum phase shift does not exceed 2π, otherwise multiple solutions may occur; Figure 7c shows that the diameter of the wire has little effect on the phase shift, but a small diameter may reduce the linearity of the resolution; Figure 7d shows that the effect of pitch on sensitivity is not obvious, and small pitch has a slight advantage in improving sensitivity.

In summary, the design of CSTLS should follow the following principles: (1). The length of the transmission line should be larger when space permits; (2). It is advisable to choose wires with larger diameter, so that the contact area with the tested liquid is larger and the sensor will be more sensitive; (3). When the mechanical strength is guaranteed, the pitch can be reduced appropriately, which ensures that a longer wire is wound in a limited space, and the sensitivity of the sensor can also be improved.

## 3. Experiment

**Figure 8:**
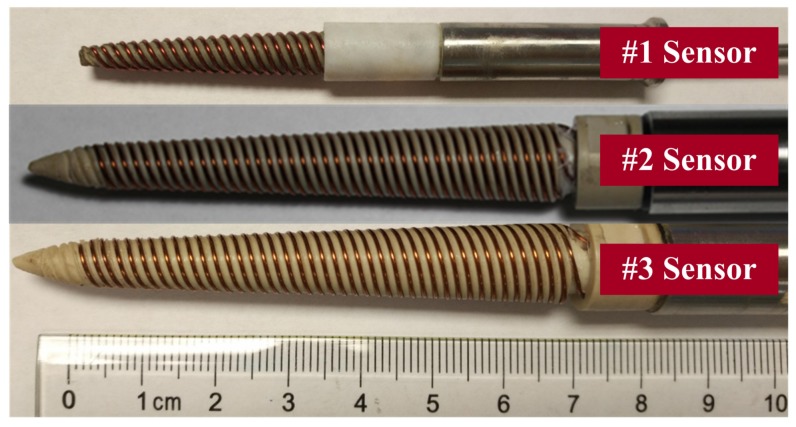
The three kinds of CSTLS.

According to theoretical analysis, three sensors with different sizes are designed. The actual sensors are shown in Figure 8. The #1 sensor is the shortest, and the length of its detection part is only 30 mm. Sensors #2 and #3 have the same length but with different enameled wire diameters. All three sensors are made with the same pitch. The specific parameters are shown in Table 3.

### 3.1. Detection Circuit and Basic Principle

**Figure 9:**
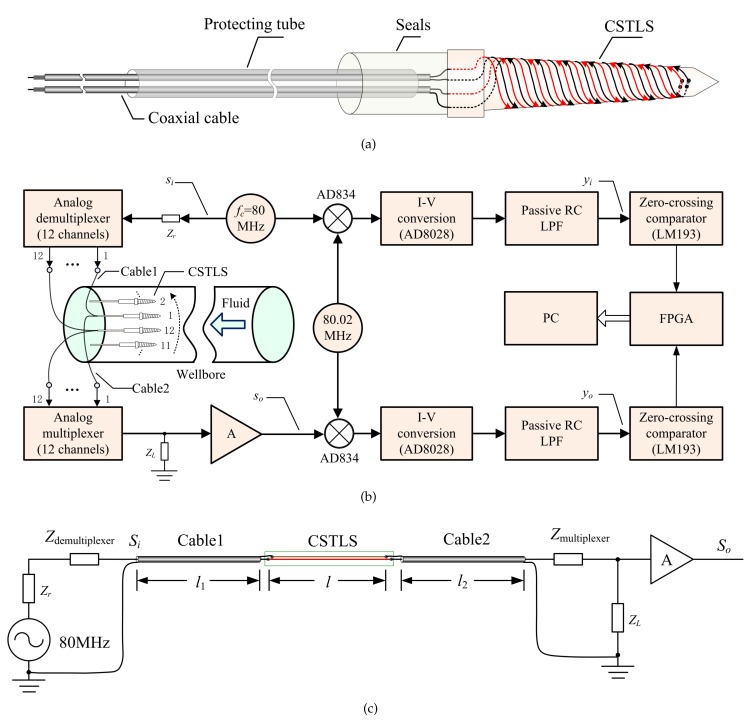
Detection circuit schematic and equivalent circuit model: (**a**) CSTLS connection schematic; (**b**) Detection circuit schematic; (**c**) Equivalent circuit model of the transmission line.(RC: resistor and capacitor; LPF: low pass filter; FPGA: field programmable gate array; PC: personal computer)

The Detection circuit schematic and equivalent circuit model is shown in Figure 9. In Figure 9a, two coaxial cables with a diameter of 1.17 mm are used to connect the CSTLS and the detection circuit. The detection circuit consists of a high-frequency signal source, a demultiplexer and a multiplexer, and a mixer circuit, as shown in Figure 9b. The 12-channel demultiplexer distributes the 80 MHz high frequency signal to 12 sensors in a time-sharing manner, and the 12-channel multiplexer multiplex the output of 12 sensors into the same channel in turn.

Suppose the input signal of the transmission line is $s_{i}\left(t\right)=A\sin\left(2\pi f_{c}t\right)$, according to the equivalent model of Figure 9c, assuming that the phase shift generated after the signal passes through cable1, CSTLS and cable2 successively is $\Phi_{1}$, Φ, $\Phi_{2}$, respectively, and the total phase shift is $\Phi_{sum}$, then, $\Phi_{sum}=\Phi_{1}+\Phi +\Phi_{2}$, the signal at the output end of the transmission line can be expressed as $s_{o}\left(t\right)=B\sin\left(2\pi f_{c}t-\Phi_{sum}\right)$.

This is a multi-branch transmission line model. The lengths of cable1 and cable2 ($l_{1}$, $l_{2}$), as well as the series impedance $Z_{r}$, and load impedance $Z_{L}$ will affect $\Phi_{sum}$, which is more complex than the single transmission line model. Therefore, we need to carefully adjust these four parameters to expect the dynamic of $\Phi_{sum}$ to be the maximum when CSTLS is tested in samples with a water holdup of 0% pure oil to 100% pure water, which means the maximum resolution is obtained. We typically also need to take into account the linearity of the curve. In summary, once these parameters are adjusted, $\Phi_{1}$ and $\Phi_{2}$ are fixed constants, and the value of $\Phi_{sum}$ indirectly reflects the value of Φ. Therefore, we transform the detection of water holdup into the measurement of the phase shift difference between two high frequency signals $s_{i}\left(t\right)$ and $s_{o}\left(t\right)$.

For accurate measurement, the method of differential frequency phase shift measurement is adopted. The basic principle is as follows. Multiply $s_{i}\left(t\right)$ and $s_{o}\left(t\right)$ by a standard sinusoidal signal $p\left(t\right)=\sin[2\pi \left(f_{c}+▵f\right)t]$, where $▵f<<f_{c}$, then:

(23) $$p\left(t\right)s_{i}\left(t\right)=A\sin\left[2\pi \left(f_{c}+▵f\right)t\right]\sin\left(2\pi f_{c}t\right)$$

(24) $$p\left(t\right)s_{o}\left(t\right)=B\sin\left[2\pi \left(f_{c}+▵f\right)t\right]\sin\left(2\pi f_{c}t-\Phi_{sum}\right).$$

According to the trigonometric formula, there is

(25) $$p\left(t\right)s_{i}\left(t\right)=0.5A\cos\left[2\pi ▵ft\right]-0.5A\cos\left[2\pi \left(2f_{c}+▵f\right)t\right]$$

(26) $$p\left(t\right)s_{o}\left(t\right)=0.5B\cos\left[2\pi ▵ft+\Phi_{sum}\right]-0.5B\cos\left[2\pi \left(2f_{c}+▵f\right)t-\Phi_{sum}\right].$$

Filter out the high frequency part of the above two equations, and retain the low frequency part, then

(27) $$y_{i}\left(t\right)=0.5A\cos\left(2\pi ▵ft\right)$$

(28) $$y_{o}\left(t\right)=0.5B\cos\left(2\pi ▵ft+\Phi_{sum}\right).$$

The above equations show that although the phase shift does not change, the frequency decreases greatly due to $▵f<<f_{c}$ (▵f = 20 KHz), which enhances the time-domain measurability. It can be seen that the delay corresponding to the phase shift of the 20 KHz signal is 4000 times larger than that of the 80 MHz signal. For the two-channel square wave signal with 20 KHz output by the zero-crossing comparator, the period is 50 μs. If a counter is used and the resolution is 1‰, the counting clock period should not exceed 50 ns. In other words, the working frequency of the counter should not be less than 20 MHz. In the actual circuit, we use a counter with a clock frequency of 40 MHz, which means that the resolution of the counter reaches 0.5‰. The circuit can divide the signal with a period of 12.5 ns into 2000 units, and the time resolution is 0.625 ps.

In order to achieve good electromagnetic compatibility (EMC) and stability, the circuit design follows the following principles:

1. Analog circuit and digital circuit are designed on different circuit boards. For high-frequency circuit and low-frequency circuit on analog circuit boards, separate wiring should be carried out according to region division to minimize the interference between them.
2. Different from the low-frequency circuit, the grounding wire of the high-frequency circuit should be large-area copper coating, nearby grounding or multi-point grounding. The ground wire should be short and thick, so as to minimize the impedance of the ground circuit and thus reduce electromagnetic interference.
3. In order to reduce the size of the circuit board, choose electronic components with small volume and high integration as far as possible. In order to make the circuit work normally in high temperature environments, we should select components with high temperature characteristics.

**Figure 10:**
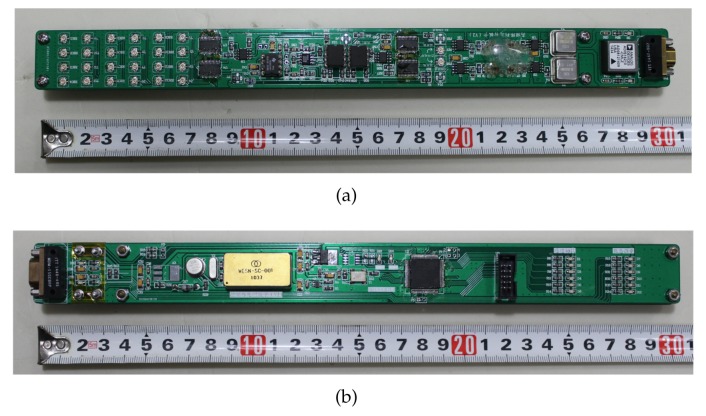
Circuit board in the tool based on signal phase shift on the transmission line: (**a**) High-frequency signal transmitting and receiving circuit board; (**b**) Signal processing and communication circuit board.

There are two circuit boards in the tool, as shown in Figure 10. Figure 10a is the high frequency signal transmitting and receiving circuit board, which is mainly composed of a temperature-compensated crystal oscillator, LC filter circuit, demultiplexer, multiplexer and mixer. Figure 10b shows the signal processing and communication circuit board, which is mainly composed of a FPGA minimum system, communication modulation and demodulation circuit, and high-precision triaxial inclinometer. The two boards have been aged at 175 ${}^{\circ }$C for 10 h.

### 3.2. Sensor Resolution Experiment

**Figure 11:**
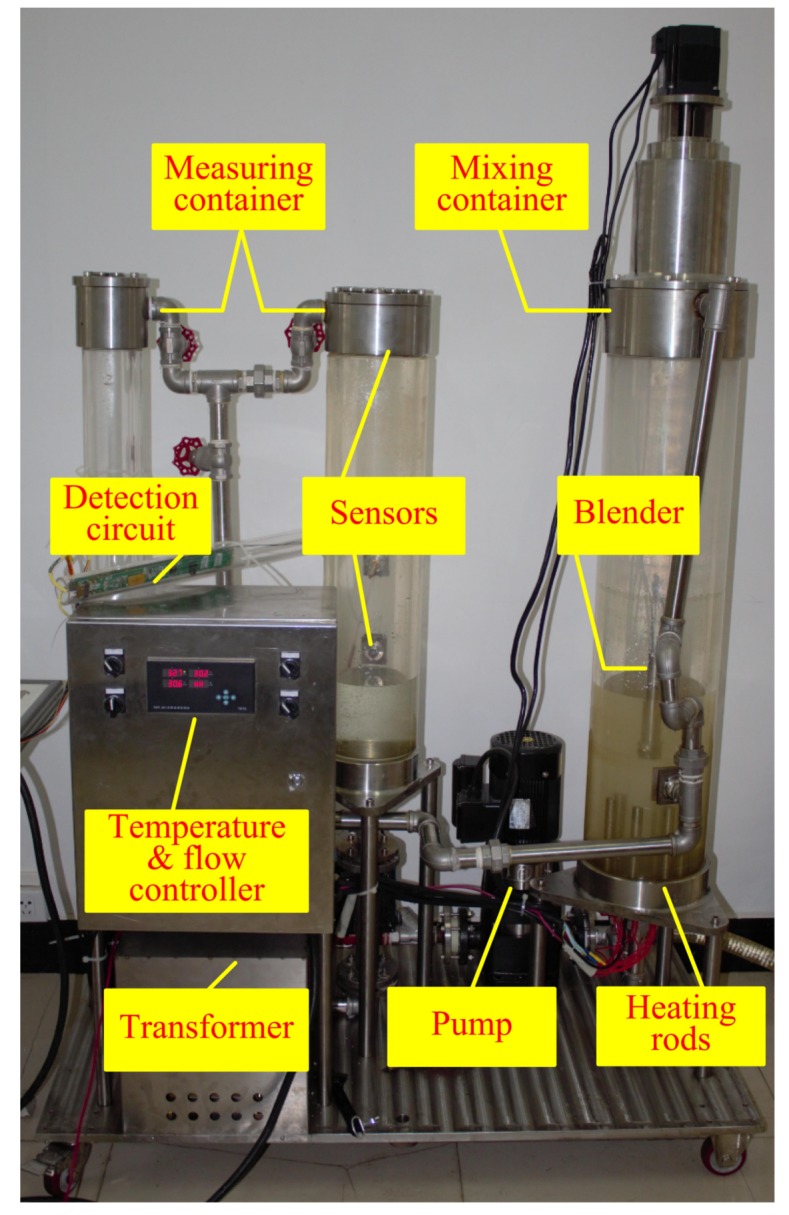
Experimental device.

In order to detect the resolution of the CSTLS, we designed an experimental device as shown in Figure 11. The prepared mixture of diesel and water is poured into the mixing container and stirred into a uniform emulsion flow with a blender. The CSTLS is fixed on the top or side of the measuring container, and the interface is connected to the detection circuit through a coaxial cable. During the experiment, the temperature was kept at about 30 ${}^{\circ }$C. The phase shift of three sensors was recorded by 21 oil–water samples with water holdup ranging from 0 to 100% and an interval of 5%. During the experiment, since the accuracy of the measurement system and the uniformity of oil–water mixing will change with time, in order to better evaluate the overall performance of the sensor and measurement system, the sampling frequency of the system is 10 times/second, and data were recorded continuously for 60 s for each different sample. In data processing, we will average 600 data and regard the average as the response value of the sensor in the sample. Meanwhile, we use the error bars to indicate the magnitude of the error.

**Figure 12:**
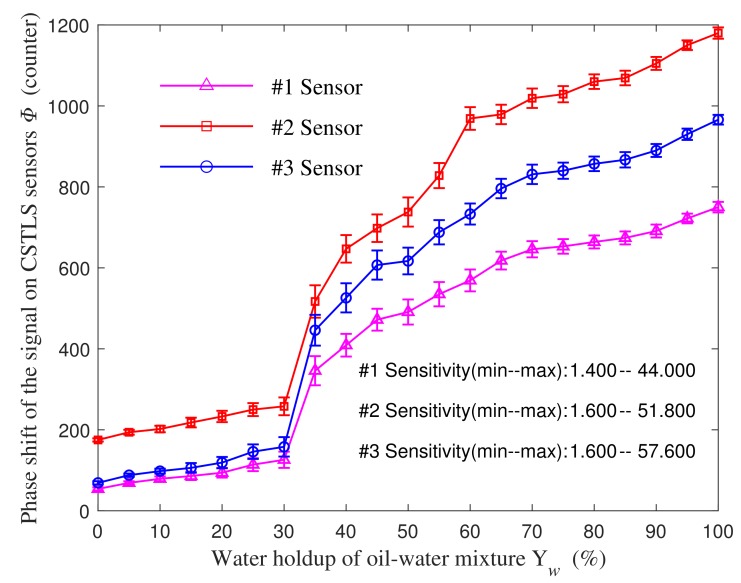
Experimental results of CSTLS.

Figure 12 shows the signal phase shift of three sensors in different samples. The conclusions are as follows: (1). The signal phase shift of CSTLS sensor has a good monotonically increasing relationship with water holdup. They are close to a linear relationship when the water holdup is 0~30% and 70~100%. The sensor sensitivity reaches its maximum when the water holdup is 30~40%, which may be related to the oil–water mixing state. (2). The length difference between the #1 and #2 sensors indicates that a longer sensor (#2) has a higher resolution, and the diameter difference between #2 and #3 sensors shows that larger diameter sensor (#2) has a higher resolution. These are consistent with previous simulation results. (3). The signal transmitter and the receiver of the CSTLS are on the same side, different from the structure of the transmitter and the receiver of the coaxial transmission line sensor on both sides, which is more conducive for wiring and sealing, and reduces the difficulty of engineering implementation.

**Figure 13:**
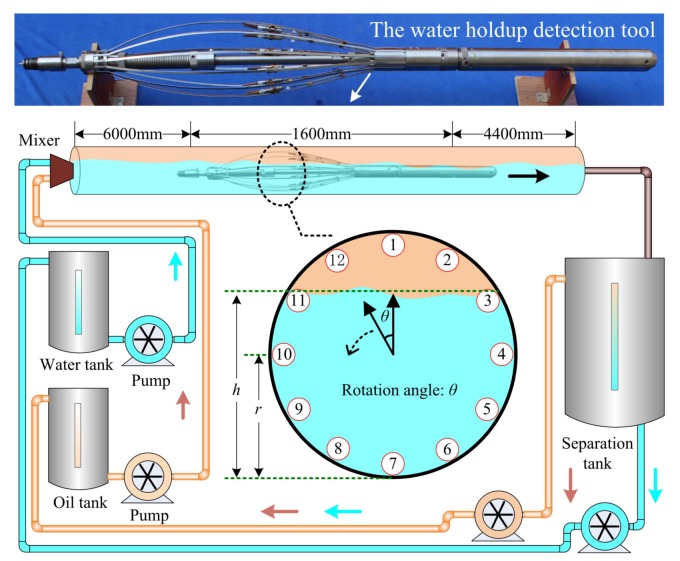
Schematic diagram of a horizontal well experimental device.

Although the dynamic range of the #1 sensor is not the largest, the #1 sensor is the smallest of the three sensors. The sensitivity of #1 sensor is 1.4~44, and the unit is counting number/1% water holdup. This means that the counter in the measuring system changes by at least 1.4 units when the water holdup of the measured oil–water mixture changes by 1%. Considering the noise of the measurement system and the influence of the actual logging environment, we conservatively believe that the measurement system has a full-scale resolution better than 3%. Therefore, we decided to use 12 sensors of this type to design the sensor-array water holdup detection tool. According to the electronic characteristics of the sensor, we developed a multi-channel detection circuit and assembled a CSTLS-based sensor-array water holdup detection tool, as shown in Figure 13. The tool length is 1600 mm, with 12 spring-fixed sensor array in the middle. When the spring sheet is closed, the tool diameter is about 43 mm, ensuring the smooth passage of the tubing in the moving mode. When the spring sheet is opened, the maximum tool diameter is about 180 mm. Owing to the spring sheet tension, the sensor is close to the inner wall of the casing.

### 3.3. Simulating a Horizontal Well Experiment

In order to test the overall performance of the tool, a test was carried out on the oil-gas-water three-phase flow experimental device at Yangtze university. The experimental device consists of an oil–water circulation system, control system and simulated casing, as shown in Figure 13.

#### 3.3.1. Experimental Parameters and Procedure

We use #10 industrial white oil and tap water as the experimental media. The oil density is 0.856 g/cm${}^{3}$ and the water density is 1 g/cm${}^{3}$. By adjusting the flow rates of the oil and the water pumps, flow conditions with water cut (100%, 80%, 60%, 40%, 20%, and 0%) can be obtained. By adjusting the angle of the simulated casing, any deviation from the vertical well to the horizontal well can be obtained. The simulated casing is 12 m long plexiglass with an outer diameter of 140 mm (5.5 in.) and an inner diameter of 120 mm (4.7 in).

Since the responses of each sensor on the tool are slightly different, the tool should be calibrated before the experiment. For each sensor, the tool scans 10 times per second, and records about 60 s in each well condition. Since the stratified (ST) flow is more representative, the following analysis is based on the measurement results when the flow rate is 20 m${}^{3}$/d and ST flow. In order to obtain the actual water holdup in the simulated casing, the position of sensors and the current distribution of oil and water in the casing were photographed with a digital camera, and the height of the oil–water interface was recorded.

#### 3.3.2. Data Analysis Method

1. Calculation of the actual water holdup in casing.

①Assuming that the height of the oil–water interface in the casing is *h* and the inner radius of the casing is *r*, according to the geometric principle, the ratio of the bow area formed by the water below the interface to the whole circular area is the actual water holdup in the casing, $Y_{real}$, which can be expressed as [31,32]

(29) $$Y_{real}=\left\{\begin{array}{cc} \frac{\arccos\left(\left(r-h\right)/r\right)r^{2}-\left(r-h\right)\sqrt{2rh-h^{2}}}{\pi r^{2}} & h<r\\ 0.5 & h=r\\ \frac{\arccos\left(\left(r-h\right)/r\right)r^{2}+\left(r-h\right)\sqrt{2rh-h^{2}}}{\pi r^{2}} & h>r. \end{array}\right.$$

2. Calculation of the water holdup in casing based on tool measurement results.

① Distance Inverse Ratio Weighted Interpolation (DIRWI) Method

Assuming that $P_{i}\left(x_{i},y_{i}\right)$ is the point coordinate on the casing section, $D_{ij}$ is the distance from the $i_{th}$ sensor point to the non- sensor point $P_{j}\left(x_{i},y_{i}\right)$ on the casing section, $Y_{i}$ is the water holdup measured by the ith sensor, and $Y_{j}$ is the predicted water holdup at the non-probe point $P_{j}\left(x_{i},y_{i}\right)$ on the casing section. The principle of DIRWI is that the correlation between points on the casing section decreases continuously with the increase of distance. The weight coefficient is related to the distance between the insertion point and the sensor in the region, and is the reciprocal of the square of the distance. The predicted water holdup is the weighted sum of all the sensor measurements in the casing area [33,34]. Namely:

(30) $$Y_{j}=\frac{\sum_{i=1}^{12}\frac{Y_{i}}{D_{ij}^{2}}}{\sum_{i=1}^{12}\frac{1}{D_{ij}^{2}}}.$$

② Gauss Radial Basis Function Interpolation (GRBFI) Method.

Gaussian radial basis function is a weight coefficient calculation function related to the distance with continuous first-order and second-order reciprocals. Its characteristic is that the influence degree of the source points on the surrounding points decreases steadily with the increase of distance. When the influence degree of source point on the surrounding points exceeds a certain range, it decreases rapidly with the increase of distance. This characteristic is consistent with the structure characteristics of the sensor-array water holdup detection tool. The Gaussian radial basis function ϕ(r) is

(31) $$\phi \left(r\right)=e^{\frac{-r^{2}}{\sigma_{ij}^{2}}}$$

where, *r* is the distance between the point to be inserted and the sensor, $\sigma_{ij}$ is the decreasing control coefficient between the two points *i*, *j*.

The predicted water holdup of the interpolated points on the casing section is [35]

(32) $$Y_{j}=\sum_{i=1,i\neq j}^{12}Y_{i}\cdot e^{\frac{-[{\left(x_{i}-x_{j}\right)}^{2}+{\left(y_{i}-y_{j}\right)}^{2}]}{\sigma_{ij}^{2}}}.$$

#### 3.3.3. Experimental Results

In order to visually reflect the water holdup measured by each sensor, the results ofthe normalized detection values are displayed in pseudo-color. A palette with a gradual change from dark brown to pale yellow is designed, corresponding to the change of water holdup in the range of 0~100%. Dark brown indicates crude oil with 0% water holdup, while pale yellow represents 100% water holdup. Therefore, a mapping table from water holdup to the pseudo-color is established. When the water holdup changes, the color of the sensor image changes accordingly.

**Figure 14:**
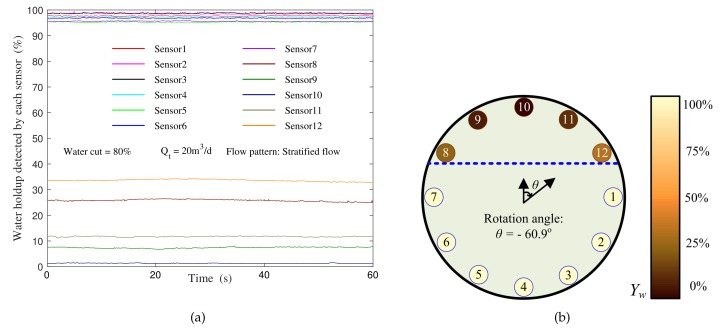
The sensors response: (**a**) Response curve; (**b**) Sensor distribution and the oil–water interface.

Figure 14a shows the water holdup curve measured by each sensor under the condition of water cut 80%. The precision triaxial inclinometer in the tool shows that the No. 12 sensor is deflected by -60.9 from the default direction, so the No. 10 sensor is moved 0.9 clockwise above the tool. Therefore, according to the sensor response and the inclinometer indication, we can obtain the sensor distribution and the oil–water interface as shown in Figure 14b.

**Figure 15:**
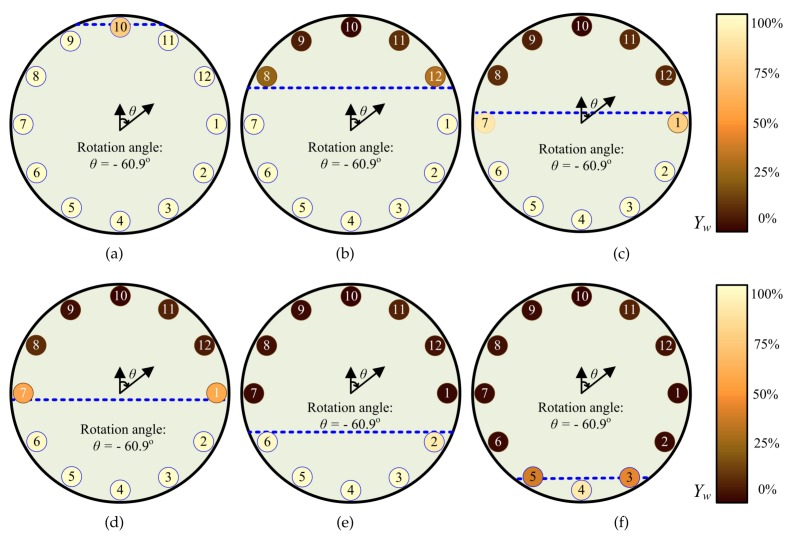
The distribution of oil and water in casing reconstructed from the measured data(horizontal well/ inclination: 90, flow rate: 20m${}^{3}$/d): (**a**) Water cut 100%; (**b**) Water cut 80%; (**c**) Water cut 60%; (**d**) Water cut 40%; (**e**) Water cut 20%; (**f**) Water cut 0%.

On this basis, we changed the water cut in the casing and obtained a number of experimental data as shown in Figure 15.

According to the water holdup measured by the sensor and the orientation information of the tool under current well condition, the measured water holdup can be calculated by the DIRWI method and the GRBFI method. The difference between the measured and the actual water holdup is the tool measurement error, as shown in Table 4.

**Figure 16:**
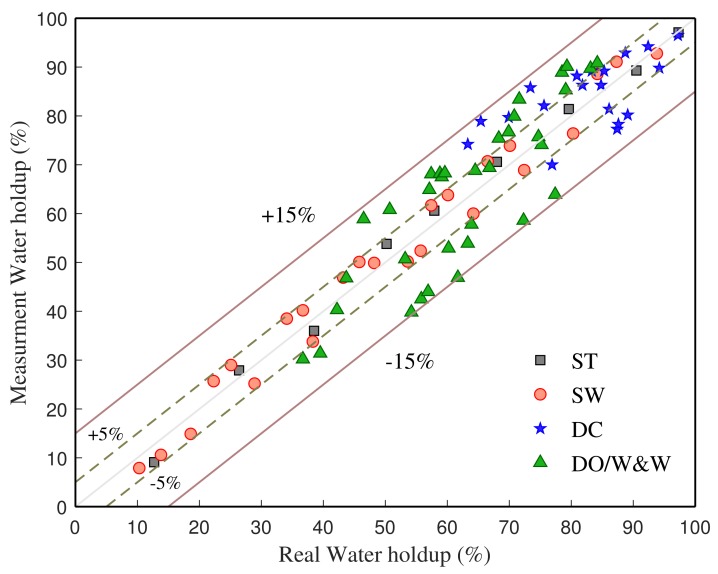
The measurement results of the tool under different flow patterns.

In order to investigate the applicability of the tool under different flow patterns, we conducted experiments under three other flow patterns: stratified wavy flow (SW), dual continuous flow (DC), and dispersed oil-in-water and water flow (DO/W&W). The responses of array CSTLS under different flow patterns were recorded experimentally. Figure 16 shows the measurement results of the tool under different flow patterns. The average relative errors for the water holdup prediction are 2.63%, 3.58%, 6.69% and 11.21% for ST, SW, DC and DO/W&W flow, respectively. The results indicate that the CSTLS-based sensor-array water holdup detection tool is effective in measuring the water holdup of horizontal oil–water two-phase flow. We can see that in the quiet ST flow, the tool responses show good linearity and minimum measurement error and reflect the position of the oil–water interface clearly. In the SW flow, although some of the sensors are sometimes immersed in oil and sometimes in water, we can still obtain relatively small measurement errors by integration. Although the flow stuctures of DC and DO/W&W are extremely nonuniform, the tool response still shows good linearity and sensitivity to water holdup changes.

## 4. Discussion

1. The basic principle of water holdup measurement based on the transmission line method is discussed in detail. The results show that the phase shift increases monotonously while the amplitude changes non-monotonously with the increase of the water holdup of the fluid as the filling medium between transmission lines. Therefore, it is feasible to measure the water holdup through the phase shift characteristics, but not through the amplitude characteristics.

2. Four common transmission lines are analyzed, and a novel design scheme of water holdup sensors based on a conical spiral transmission line is proposed. The sensor has a size of 30 mm × 6 mm (height × cone base diameter). Its volume is 0.28 cm${}^{3}$, only 1/160 of the current coaxial transmission line sensor volume, and only 1/3 of the current smallest capacitance and conductance water holdup sensors, realizing the senor miniaturization.

3. The transmission line method is an effective method to measure water holdup in the whole range of 0%~100%. By using the CSTLS-based sensor-array water holdup detection tool, we can get less than 3.6% measurement error in the ST and SW flows, but more than 5% measurement error in DC and DO/W&W flows, which may be related to fluid nonuniformity. In addition, we currently calculate the water holdup of a single probe by averaging the response over a period of time. This may not be the most reasonable data processing method for uneven flow patterns, and we need to improve the algorithm in subsequent studies.

4. Temperature will change the dielectric constant of oil–water mixture and salinity will change liquid conductivity. Therefore, how to reduce the measurement error caused by temperature and salinity will be the future research direction.

## 5. Conclusions

To measure the water holdup in horizontal wells, we analyzed the water holdup measurement theory based on transmission line and proposed an innovative design method of water holdup sensors based on conical spiral transmission lines. By winding parallel double lines on a spiral cone, a longer length is achieved in a limited space, which solves the problem that traditional coaxial transmission lines are easy to block and not easy to miniaturize. Moreover, the conical spiral structure reduces the adhesion of liquid surface tension effectively, contacts the measured liquid directly, and finally, improves the resolution of detection to 3%. Our developed CSTLS-based sensor-array water holdup detection tool achieves full range (0%~100%), array (12-point), and high precision (3.6% error in ST and SW flow) water holdup detection.

## 6. Patents

Chen, Q.; Liu, G.; Yu, H.; Guo, Y.; Wei, Y.; Qu, F. The invention relates to a cylindrical water holdup detection probe based on electromagnetic waves. China Patent 201610663697. X, 12 December 2016.

Wei, Y.; Yu, H.; Liu, G.; Chen, Q.; Zhou, Y. The downhole crude oil water holdup detect device based on coplanar microstrip transmission line. China Patent 2439512, 5 April 2017.

## Figures and Tables

**Table 1:** Relationship between the characteristic impedance and the phase shift factor of the four kinds of transmission lines and the dielectric constant of the filled medium.

Parameter	Parallel Line	Coaxial Cable	Microstrip	Coplanar Microstrip Lines
$Z_{0}$	$\frac{\mathrm{acosh}\left(D/d\right)\sqrt{\mu }}{\pi \sqrt{\varepsilon_{0}\varepsilon_{r}}}$	$\frac{\ln\left(b/a\right)\sqrt{\mu }}{2\pi \sqrt{\varepsilon_{0}\varepsilon_{r}}}$	$\frac{s\sqrt{\mu }}{w\sqrt{\varepsilon_{0}\varepsilon_{r}}}$	$\frac{30\pi k}{\sqrt{\varepsilon_{0}\varepsilon_{eff}}}$
β	$\omega \sqrt{\mu \varepsilon_{0}\varepsilon_{r}}$	$\omega \sqrt{\mu \varepsilon_{0}\varepsilon_{r}}$	$\omega \sqrt{\mu \varepsilon_{0}\varepsilon_{r}}$	$k\omega \sqrt{\mu \varepsilon_{0}\varepsilon_{eff}}$

$\mu ,\varepsilon_{r}$—- Permeability, relative permittivity of the medium around conductor; $\varepsilon_{eff}$ —-The effective dielectric constant of the transmission line; *k*—-The structure constant of coplanar microstrip lines; $\varepsilon_{0}$ —-The dielectric constant of air.

**Table 2:** Default simulation parameters table of the CSTLS.

Parameter	Equivalent Length	Wire Diameter	Pitch	Signal Frequency	Load Impedance
Value	400 mm	0.80 mm	2.00 mm	80 MHz	60 Ω

**Table 3:** Specific parameters of the three kinds of CSTLS.

CSTLS	Equivalent Length	Wire Diameter	Pitch	Number of Turns
#1	192 mm	0.78 mm	2.00 mm	22
#2	544 mm	0.78 mm	2.00 mm	42
#3	544 mm	0.49 mm	2.00 mm	42

**Table 4:** Measured and actual water holdup, and error by using different calculate method. Distance inverse ratio weighted interpolation (DIRWI), and Gauss radial basis function interpolation (GRBFI) methods.

Water	Response Values of Sensors						Actual	DIRWI Method		GRBFI Method	
Cut	No. 1–12 (Normalized: %)						$Y_{\mathrm{real}}$	$Y_{\mathrm{pre}}$	Error	$Y_{\mathrm{pre}}$	Error
100%	98.4	99.1	98.9	98.9	98.7	97.8	97.3%	97.1%	−0.2%	96.1%	−1.2%
	96.3	97.2	94.2	75.3	93.5	98.8					
80%	97.7	98.5	98.8	97.1	95.3	96.8	68.0%	70.6%	2.6%	66.2%	−1.8%
	95.6	25.8	7.4	1.3	11.7	33.6					
60%	76.5	97.4	98.7	99.0	98.5	97.3	57.9%	60.6%	2.7%	58.5%	0.6%
	78.4	11.2	7.8	0.9	10.7	9.7					
40%	58.8	97.6	98.7	98.8	98.5	97.5	50.2%	53.8%	3.6%	53.2%	3.0%
	55.9	8.6	3.6	1.1	8.5	6.7					
20%	1.7	91.3	98.5	98.8	98.5	97.5	38.5%	36.0%	−2.5%	39.8%	1.3%
	2.3	3.9	2.4	0.7	8.7	4.5					
0%	4.2	3.7	44.6	89.5	40.1	4.5	12.7%	9.1%	−3.6%	14.0%	1.3%
	1.9	1.7	2.5	1.1	6.4	3.3					

## References

[1] Miller C., Waters G., Rylander E. (2011). Evaluation of production log data from horizontal wells drilled in organic shales. Proceedings of the North American Unconventional Gas Conference and Exhibition.

[2] Ryan N., Hayes D. (2001). A new multiphase holdup tool for horizontal wells. Proceedings of the SPWLA 42nd Annual Logging Symposium.

[3] Zett A., Webster M., Noordermeer A., Hockley M., Lockyer G., Browne H., Donkin C. (2012). New sensor development helps optimize production logging data acquisition in horizontal wells. Petrophysics.

[4] Frisch G., Jung M., Alldredge P., Zett A., Webster M. (2009). Providing accurate PI interpretation with multi-probe, multi-sensor tools in segregated flow environments. Proceedings of the SPWLA Annual Logging Symposium.

[5] Zhai L., Jin N., Zong Y., Gu M. (2012). The development of a conductance method for measuring liquid holdup in horizontal oil–water two-phase flows. Meas. Sci. Technol..

[6] Kong W., Kong L., Li L., Liu X., Xie R., Li J., Tang H. (2016). The finite element analysis for a mini-conductance probe in horizontal oil–water two-phase flow. Sensors.

[7] Zhai L., Zhang H., Yan C., Jin N. (2018). Measurement of oil–water interface characteristics in horizontal pipe using a conductance parallel-wire array probe. IEEE Trans. Instrum. Meas..

[8] Wang D., Jin N., Zhai L., Ren Y. (2019). Characterizing flow instability in oil-gas-water three-phase flow using multi-channel conductance sensor signals. Chem. Eng. J..

[9] Lam L., William P., Nor H., Tong T. (2016). Design of helical capacitance sensor for holdup measurement in two-phase stratified flow: A sinusoidal function approach. Sensors.

[10] Li L., Kong L., Xie B., Fang X., Kong W., Liu X., Wang Y., Zhao F. (2019). The Influence on response of a combined capacitance sensor in horizontal oil–water two-phase flow. Appl. Sci..

[11] Strazza D., Demori M., Ferrari V., Poesio P. (2011). Capacitance sensor for hold-up measurement in high-viscous-oil/conductive-water core-annular flows. Flow Meas. Instrum..

[12] Zhang H., Zhai L., Yan C., Wang H., Jin N. (2018). Capacitive phase shift detection for measuring water holdup in horizontal oil–water two-phase flow. Sensors.

[13] Ishimaru A. (2017). Electromagnetic Wave Propagation, Radiation, and Scattering: From Fundamentals to Applications.

[14] Guo H., Wu X., Jin Z., Zhao H. (1993). The design and development of microwave holdup meter and application in production logging interpretation of multiphase flows. Proceedings of the SPE Annual Technical Conference and Exhibition.

[15] Wang J., Qiang X., Zhang Y. (2002). Coaxial transmission line phase method for measuring water content of oil well. Chin. J. Sci. Instrum..

[16] Meng X., Wang J. (2005). Optimized design of the coaxial line sensor. J. Daqing Pet. Inst..

[17] Garcìa-Baños B., Catalá-Civera J., Canós A., Peñaranda-Foix F. (2005). Design rules for the optimization of the sensitivity of open-ended coaxial microwave sensors for monitoring changes in dielectric materials. Meas. Sci. Technol..

[18] Kapilevich B., Litvak B. (2014). Microwave measurements of dielectric properties using a gap-coupled multi-mode coaxial resonator. Measurement.

[19] Yu H., Wei Y., Tang T., Liu G. (2012). Theoretical analysis of measuring dielectric constant of oil–water mixture based on electromagnetic wave on coaxial line. Well Logging Technol..

[20] Wei Y., Yu H., Chen Q., Tang T., Liu G., Qu F. (2012). Research and experiment on electromagnetic wave water holdup sensor. Transducer Microsyst. Technol..

[21] Huo X., Sun W., He F., Yang Z., Peng Y. (2014). Coupling analysis of low-speed multiphase flow and high-frequency electromagnetic field in a complex pipeline structure. Math. Probl. Eng..

[22] Peng Y., Sun W., Huo X., Yang Z. (2013). Water holdup measurement method based on microwave transmission line principle. J. Oil Gas Technol..

[23] Mohamed A., Elgamal M., Said R. (2006). Determination of water content and salinity from a producing oil well using cpw probe and eigendecomposition. Sens. Actuators A Phys..

[24] Wei Y., Yu H., Dai J., Liu G., Chen Q. (2017). Water holdup measurement of oil–water two-phase flow based on CPW. Chin. J. Sci. Instrum..

[25] Wei Y. Study on Water holdup detection method and downhole tools development of oil–water two-phase flow based on multiple transmission lines. Proceedings of the 3rd International Conference on Gas, Oil and Petroleum Engineering (GOPE-2019).

[26] Wei Y., Yu H., Chen Q., Liu G., Chen J. (2019). Measurement of water holdup in oil–water two-phase flows using coplanar microstrip transmission lines sensor. IEEE Sens. J..

[27] Chen J. (2015). A semianalytical finite element analysis of electromagnetic propagation in stratified media. Microw. Opt. Technol. Lett..

[28] Wang F., Ho S.C.M., Huo L., Song G. (2018). A novel fractal contact-electromechanical impedance model for quantitative monitoring of bolted joint looseness. IEEE Access.

[29] Chen J., Liu Q. (2012). Discontinuous Galerkin time-domain methods for multiscale electromagnetic simulations: A review. Proc. IEEE.

[30] Luo M., Li W., Wang J., Wang N., Chen X., Song G. (2018). Development of a novel guided wave generation system using a giant magnetostrictive actuator for nondestructive evaluation. Sensors.

[31] Zhai L., Jin N., Zong Y., Hao Q., Gao Z. (2015). Experimental flow pattern map, slippage and time–frequency representation of oil–water two-phase flow in horizontal small diameter pipes. Int. J. Multiph. Flow.

[32] Zhang H., Zhai L., Han Y., Chen X., Gao Z., Jin N. (2016). Response Characteristics of Coaxial Capacitance Sensor for Horizontal Segregated and Non-Uniform Oil–Water Two-Phase Flows. IEEE Sens. J..

[33] Frisch G., Perkins T., John Q. (2002). Integrating wellbore flow images with a conventional production log interpretation method. Proceedings of the SPE Annual Technical Conference and Exhibition.

[34] Song H., Guo H. (2016). Analysis on imaging interpolation algorithm for logging data of water holdup array tool in horizontal wells. J. Oil Gas Technol..

[35] Dai J., Guo H., Liu H., Yang Z., Yang M., Lu L. (2010). The Flow Imaging Algorithm Study on Logging Data of Capacitor Array Tool. Well Logging Technol..

